# Programming emergent symmetries with saddle-splay elasticity

**DOI:** 10.1038/s41467-019-13012-9

**Published:** 2019-11-08

**Authors:** Yu Xia, Andrew A. DeBenedictis, Dae Seok Kim, Shenglan Chen, Se-Um Kim, Douglas J. Cleaver, Timothy J. Atherton, Shu Yang

**Affiliations:** 1 Department of Materials Science and Engineering, University of Pennsylvania, Philadelphia, PA 19104 USA; 2 Department of Physics and Astronomy, Tufts University, Medford, MA 02155 USA; 3 Materials and Engineering Research Institute, Sheffield Hallam University, Sheffield, S1 1WB UK

**Keywords:** Materials science, Liquid crystals, Self-assembly

## Abstract

The director field adopted by a confined liquid crystal is controlled by a balance between the externally imposed interactions and the liquid’s internal orientational elasticity. While the latter is usually considered to resist all deformations, liquid crystals actually have an intrinsic propensity to adopt saddle-splay arrangements, characterised by the elastic constant ${K}_{24}$. In most realisations, dominant surface anchoring treatments suppress such deformations, rendering ${K}_{24}$ immeasurable. Here we identify regimes where more subtle, patterned surfaces enable saddle-splay effects to be both observed and exploited. Utilising theory and continuum calculations, we determine experimental regimes where generic, achiral liquid crystals exhibit spontaneously broken surface symmetries. These provide a new route to measuring ${K}_{24}$. We further demonstrate a multistable device in which weak, but directional, fields switch between saddle-splay-motivated, spontaneously-polar surface states. Generalising beyond simple confinement, our highly scalable approach offers exciting opportunities for low-field, fast-switching optoelectronic devices which go beyond current technologies.

## Abstract (web summary)

Liquid crystals are used for devices with increasingly complex geometries which makes it important to understand the influence of deformation. Xia et al. measure the poorly investigated saddle-splay elastic constant by means of surface patterning and simulation for programming emergent symmetries.

## Introduction

Thin liquid crystal (LC) films with controllable birefringence underpin the dominant technology utilized in flat screen and portable displays, active optical components, switches, and storage devices. This birefringence arises from a convolution of molecular-scale polarisability anisotropies and the long-range orientational order of the constituent molecules. In any device, the stable configuration is controlled by interactions with bounding substrates, applied fields, defects, and embedded components^1–5^. While multi-stability adds further functionality^6–10^, it is often associated with complex fabrication processes^11^, exquisite control of surface conditions, high threshold voltages, and slow switching^12–14^.

**Fig. 1:**
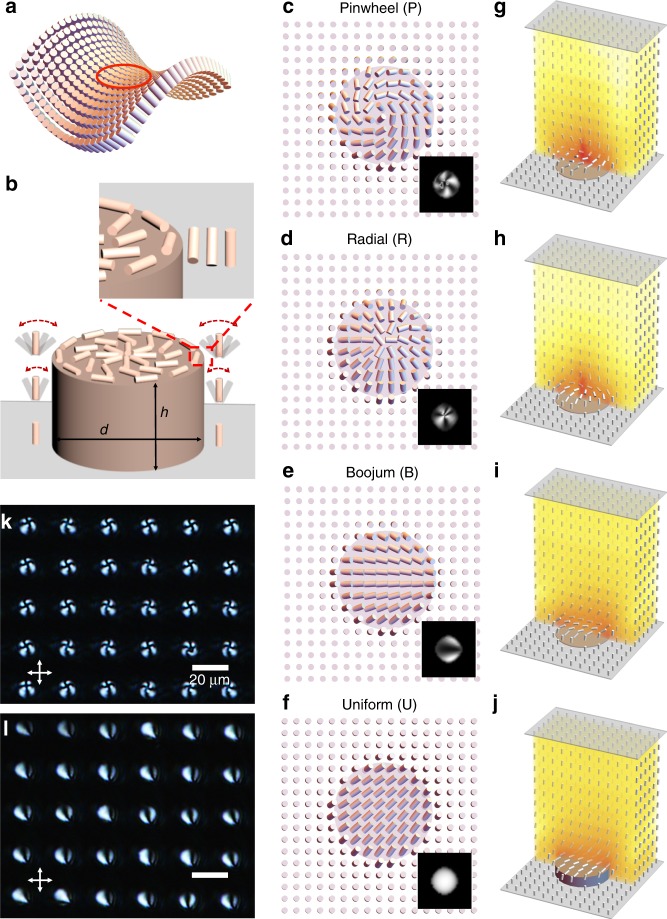
Liquid crystals on circular posts. **a** Distorted director field with saddle-splay rich region indicated in red. **b** Schematic of director reorientation over the top of a post. Liquid crystal is increasingly free to reorient with post height above the surface. **c**–**j** Stationary liquid crystal configurations with horizontal sections through the director field at the top of the post (**c**–**f**), simulated polarized optical microscope images (insets) and three dimensional reconstructions (**g**–**j**) colored by energy density. Post height is $h=0.2\ \upmu {\mathrm{m}}$ for (**c**–**e**), and $h=2\ \upmu {\mathrm{m}}$ for (**f**). **k**, **l** Experimentally observed pinwheel and boojum structures in 5CB (4-cyano-4′-pentylbiphenyl), respectively

At the continuum level, spatial variation of an LC’s orientational order is characterized by a headless vector ${\bf{n}}$, the director. The Frank-Oseen free energy^15^ measures the energy cost of different kinds of director variation in the absence of defects,

1 $$F=\frac{1}{2}\int \left[{K}_{11}{\left(\nabla \cdot {\bf{n}}\right)}^{2}+{K}_{22}{\left({\bf{n}}\cdot \nabla \times {\bf{n}}\right)}^{2}+{K}_{33}{\left|{\bf{n}}\times \nabla \times {\bf{n}}\right|}^{2} -{K}_{24}\nabla \cdot \left({\bf{n}}\times \nabla \times {\bf{n}}+{\bf{n}}\nabla \cdot {\bf{n}}\right)\right]dV$$

with four orientational elastic constants^16,17^ referred to as splay, twist, bend and saddle-splay in order of their appearance in Eq. (1). Where defects are present, the Landau-de Gennes approach must be used, although the correspondence between the elastic terms is nontrivial^15^. The first three elastic constants, ${K}_{11}$, ${K}_{22}$, and ${K}_{33}$, are readily measured and well understood. However, the final saddle-splay term, with associated constant ${K}_{24}$ (We note a number of conventions for the constant in front of the saddle-splay term exist in the literature. Here we follow^18–20^ in using a single constant *K*_24_ rather than Frank’s original notation^16^, where the prefactor appears as *K*_22_ + *K*_24_ (equal to *K*_24_ in our notation)), is quite different in character to the other three: it need not be positive and hence may promote distortion, with Ericksen’s stability requirements imposing a bound on its magnitude $0\,{<}\,\left|{K}_{24}\right|\,{<}\,2\min ({K}_{11},{K}_{22})$^21^; it is nonzero only if ${\bf{n}}$ varies in at least two orthogonal dimensions (Fig. 1a); finally, as a pure divergence, it can be integrated out to the surface of the sample and, hence, only enters the free energy through the boundary conditions^16^, although a recent reformulation retains this term in the bulk^22^.

These properties conspire to make ${K}_{24}$ notoriously difficult to measure, because a geometry is required that promotes a 3D distortion and simultaneously allows the surface anchoring energy to be measured. Hence, while the other elastic constants were measured for common LC materials decades ago, there exist to date only a handful of experimental measurements of ${K}_{24}$^18,20,23–28^, as well as pioneering attempts to compute ${K}_{24}$ directly from simulations^29,30^. However, the bounds on these experimental measurements typically exceed the range allowed by stability requirements and, hence, measuring ${K}_{24}$ to the precision of the other LC elastic constants remains an open problem.

For most LC applications, where either surface anchoring is so strong as to prevent surface distortion, or ${\bf{n}}$ depends on only one or two coordinates, saddle-splay is, quite reasonably, neglected. However, saddle-splay elasticity is also known to be capable of driving a remarkably rich phenomenology, from stabilizing textures in nematic droplets^19^ or complex geometries^31^, to promoting pattern formation in the Freedericksz transition^32^, and “lassoing” networks of line defects in a hole array^33^.

A powerful strategy for creating saddle-splay distortions in a LC director field is through surface patterning. This can be either topographic, so that curvature is induced by the varying surface normal, or chemical, such that the preferred orientation axis varies across the substrate. In the absence of applied fields, surface coupling and orientational elasticity dominate LC alignment, such that varying a surface pattern can permit full control of both the preferred bulk orientation and its effective anchoring strength^34^. Incompatibility between the surface pattern and the ordering may also promote spontaneous symmetry breaking, in which the LC adopts a surface-region configuration belonging to a subgroup of the pattern’s symmetry group. Such symmetry breaking naturally produces multiple stable configurations, which can then be used as the basis of a bi- or multistable device^11,35^.

In this paper, we combine topographic and chemical patterning to create systems that purposely accentuate configurational dependence on saddle-splay elasticity. By varying both the shape and local alignment of the patternings, and comparing details of the resulting polarized optical microscopy (POM) images with theoretical calculations, we then significantly narrow the the feasible region for ${K}_{24}$. In addition, by exploiting the resultant ability to program spontaneous symmetry breaking by design, we prototype a multistable device founded on low-voltage switching between saddle-splay-stabilized structures.

## Results

### Circular posts

Our baseline system design consists of a square array of posts with period $\lambda$, where the shape and height $h$ of the post may be varied (Fig. 1b). The tops of the posts promote planar degenerate alignment and the background surface gives vertical alignment (homeotropic anchoring). The side of each post also promotes planar degenerate alignment, compatible with the vertical anchoring where the post meets the substrate. Reorientation of the director, however, must occur at the top edge of each post. Distortion is also caused in the surface plane by curvature of the post boundaries, thereby achieving the three dimensional director variation required for saddle-splay.

We first focus on circular SU8 (an epoxy-based photoresist) posts of diameter $d=\lambda /2=10\ \upmu{\mathrm{m}}$ and height $h=0.2\ \upmu{\mathrm{m}}$, which were patterned by photolithography, followed by selective surface treatment with N,N-dimethyl-n-octadecyl-3-amino-propyltrimethoxysilyl chloride (DMOAP) (see Methods and Supplementary Fig. 1). This imposes strong homeotropic anchoring on the DMOAP-coated glass regions (see Methods), while maintaining planar alignment on the SU8 patterns. Continuum elasticity calculations were performed, as described in the Methods section, to determine the corresponding stationary director configurations for given SU8 anchoring strength $W\in [3,32]\times 1{0}^{-6}\ {\mathrm{J}}\ {{\mathrm{m}}}^{-2}$ and ${K}_{24}$, while the corresponding much stronger anchoring strength for the DMOAP substrate $W=315\ \upmu \,{\mathrm{J}}/{\mathrm{m}}^{2}$ was held constant.

**Fig. 2:**
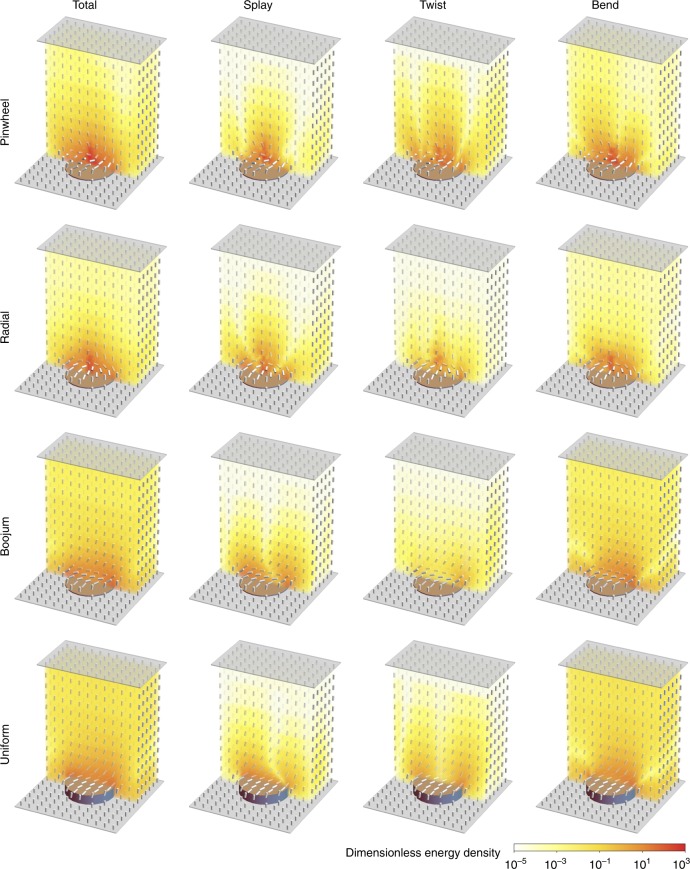
Simulated molecular alignment configurations. Calculated structures for 5CB on circular SU8 posts, colored by energy density of each elastic deformation mode. Post height is $h=0.2\ \upmu{\mathrm{m}}$ for pinwheel, radial, and boojum structures, and $h=2\ \upmu{\mathrm{m}}$ for uniform structure

The possible equilibrium arrangements are shown in Fig. 1c–e and g–i: a “pinwheel” (P) structure where the director field escapes continuously (but with spontaneous chirality) into the vertical orientation at the center of the post; a radial (R) structure with a single defect at the center of the post (in fact virtualized within it); and a boojum state (B) where a defect is localized near the post edge. For taller posts of $h=2.0\ \upmu{\mathrm{m}}$, a fourth uniform (U) state was found (Fig. 1f, j) where the director is largely uniform over the post surface and oriented along the diagonal; the other states are also stationary at this taller post height. Predicted POM images were calculated for each structure as described in Methods. These four structures represent different compromises in meeting the boundary conditions and may be favored depending on the elastic constants and anchoring energy. The key physics driving structure selection is the distortion by which the reorientation at the top edge of the post is accomplished as shown in Fig. 2 (see also Supplementary Fig. 2). We also note that for the P, R, and B states, the apparent defects are virtualized beneath the substrate on a lengthscale ${K}_{1}/W \sim 8\times 1{0}^{-7}\ {\mathrm{m}}$ due to the weak anchoring for SU8. This lengthscale is much larger than the size of a defect, on the order of a few molecular lengths, a posteriori justifying use of the Frank-Oseen energy Eq. (1) which neglects gradients in the orientational order parameter.

Crucially, the relative influence of these edge pinning lines varies with the height of the posts. This approach provides a direct experimental route for accessing the configurations identified in Fig. 1c–k. Indeed, it is this control over the effective edge pinning strength that differentiates our system from those used previously for surface-patterned LCs and, so, makes saddle-splay-stabilized configurations accessible. Further, since this experimental strategy can be extended to a variety of LC materials, surface patterns and alternative geometries, a wealth of other novel behaviors can be realized.

LC Cells were prepared with a second unpatterned DMOAP-coated substrate placed on top, closed and filled with the achiral LC, 5CB (4-cyano-4′-pentylbiphenyl) (Supplementary Fig. 1), in the isotropic state, cooled into the nematic phase, imaged by POM, and then repeatedly heated into the isotropic state and re-cooled. Two configurations, shown in Fig. 1, were observed: The first (Fig. 1k and Supplementary Fig. 3) has a $+1$ defect over each post, displays apparent breaking of achiral symmetry, and corresponds to the pinwheel (P) configuration. The second (Fig. 1l) has a defect on the edge of each post, apparently weakly aligned with the basis vector, and corresponds to the boojum (B) structure. While one structure or the other was adopted within each cooling cycle with high spatial uniformity—attesting to the uniformity of the patterning—the occurrence of P or B was apparently random with no evidence of memory between cycles. We therefore deduce that, for this system, P and B lie close in energy, with kinetic differences in each cycle promoting one or the other. The R and U structures were not observed experimentally with 5CB and 0.2 μm post height, suggesting they must be higher in energy or unstable.

### Templating with smectic phase

**Fig. 3:**
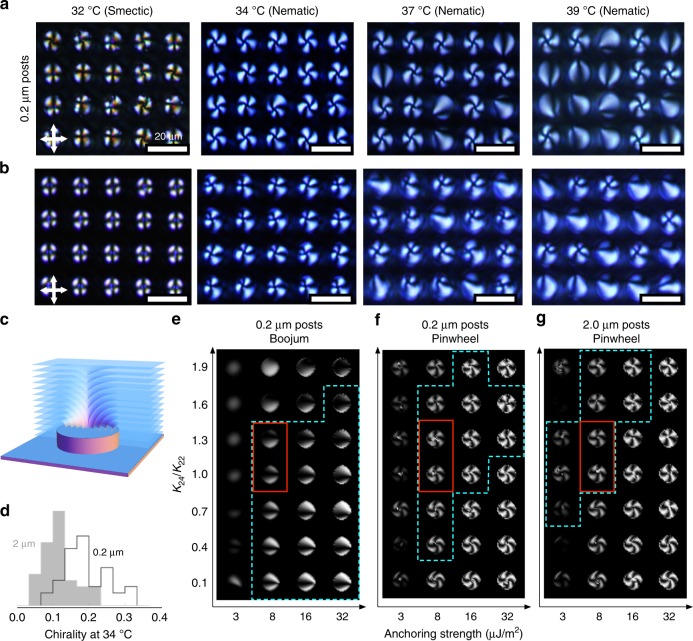
Spontaneous chiral symmetry breaking for 8CB on circular posts. POM images of 8CB on **a** 0.2 μm and **b** 2.0 μm posts at various temperatures. Both samples were slowly heated (1 °C/min) from smectic phase to nematic phase. **c** Schematic of the smectic layers adopted by 8CB on top of a SU8 post, forming a toroidal focal conic domain (TFCD). Within a TFCD, radial in-plane LC director is strongly imposed. **d** Frequency histograms of quantitative chirality measurements for 8CB at 34 °C in the nematic phase over 40 posts. The magnitude of the chirality is influenced by the post height, such that short posts induce greater chiral symmetry breaking. **e**–**g** Simulated POM images of 8CB alignment at 34 °C as a function of saddle-splay constant (${K}_{24}/{K}_{22}$) and SU8 surface anchoring strength. Blue dash boxes indicate structures consistent with experiment as described in the main text; the intersection of these three regions is highlighted in red

To investigate this control, we prepared a new cell closed and filled with another achiral LC 8CB (4-cyano-4′-octylbiphenyl) in the isotropic, and cooled to 32 °C, corresponding to the smectic phase. Now, saddle-splay deformations on the post are disallowed, and the LC adopts toroidal focal conic domains (TFCDs) above the post with uniform vertical alignment between posts (note that the smectic layers are perpendicular to the director), as shown schematically in Fig. 3c and confirmed experimentally by the POM images in Fig. 3a and b, representing different post heights. Using the TFCD arrangement as a starting condition enables us to template P or R structures where the defect is located in the center of the post, because the TFCD involves a radial in-plane LC director profile on the top of each post.

On gradual heating (1 °C/min) into the nematic phase (34 °C), local achiral symmetry breaking is observed consistently in the POM images, with left- and right-handed twists forming on different posts with equal likelihood. As there are no molecular-level chiral interactions in these systems, these twists must arise due to spontaneous onset of the saddle-splay-stabilized P structure (Fig. 1b). We take care to avoid the regime within $1-2K$ of the $N-{S}_{A}$ transition where the twist and bend elastic constants diverge^36^. We calculate, from images of each post, a measure of structural chirality as described in the Methods section below and Supplementary Fig. 4. As is evident from Fig. 3d, the magnitude of this structural chirality is sensitive to the post height: due to their reduced edge pinning strength, taller posts induce weaker chirality. On further heating, many of the pinwheels individually transition into randomly-oriented B configurations.

To fully understand these experimental observations, we return to simulation. Because, to our knowledge, there exists no experimentally measured value of ${K}_{24}$ for 8CB, we consider values across the range defined by Ericksen. Similarly, multiple values are examined for $W$, the anchoring strength of SU8 (the same strong-anchoring value ${W}_{\mathrm{DMOAP}}=315\ \upmu \,{\mathrm{J}}/{\mathrm{m}}^2$ as before is used for DMOAP). Simulations were initiated with either a +1 defect or a uniform director configuration. For all saddle-splay constants and SU8 anchoring strengths considered, the defect initial configuration consistently relaxed into P, whereas the uniform director always gave B. However, simulations that control post height and saddle-splay strength reveal smooth transitions between states with similar defects: the chirality of the P structure can unwind into the R structure, and the boojum of the B structure can escape to infinity to create the U structure. In absolute terms, these calculations show B to be the global minimum energy arrangement, consistent with the experimental observation of transitions from P on heating.

**Fig. 4:**
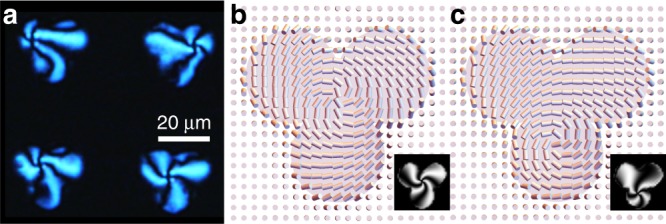
Control of defect location. **a** Polarizing microscope image of 5CB in contact with trefoil posts. **b** Section through a calculated structure with defect located at the center of the trefoil; **c** a structure with defect centered on one of the disks. Insets: Simulated POM images for each structure

Predicted POM images are displayed as a function of ${K}_{24}$ and $W$ in Fig. 3e–g. We identify regions of parameter space where simulations are consistent with experiment for each structure and post height as described in Methods and the intersection of these regions constrains ${K}_{24}/{K}_{22}$ to ${\sim} 1$ and $W$ to *W* = 8 μJ/m^2^. Indeed, given the strong sensitivity of these structures to ${K}_{24}$, we suggest that measurement of pinwheel features such as these could be a viable route to determining the splay-bend elastic constant for a range of LC materials. Further, as demonstrated in the supplementary material, we can also exploit our ability to sculpt posts of arbitrary cross section to control the location of the defects and observe multistable behaviors using, e.g., trefoil (Fig. 4), square and quatrefoil posts (Supplementary Fig. 5); here defects reside in the post centers or on one of the patterned lobes.

### Annular posts

**Fig. 5:**
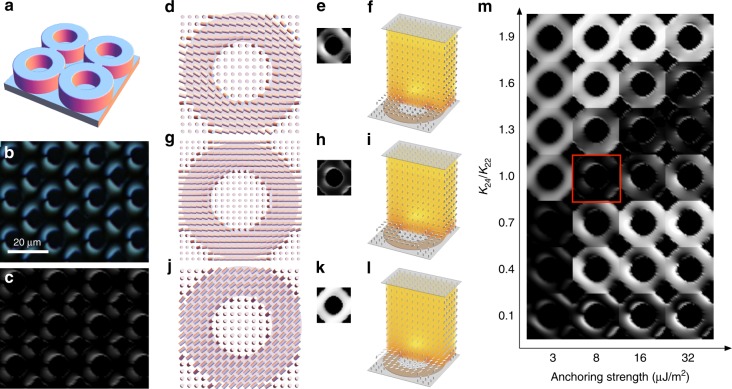
Periodic annular posts. **a** Schematic of annuli pattern, showing several periods. **b** Experimental observation and **c** simulation of 5CB on an array of touching annuli. An extended array of −1/2 defects forms and breaks the pattern symmetry. **d**–**l** Calculated director profiles (**d**, **g**, **j**) at the top of post, (**e**, **h**, **k**) simulated POM images and (**f**, **i**, **l**) director profiles throughout the the cell for *W* = 8 μJ/m^2^ and **d**–**f** ${K}_{24}/{K}_{22}=0.7$, **g**–**i**${K}_{24}/{K}_{22}=1$ (the value that best agrees with experiment) and **j**–**l** ${K}_{24}/{K}_{22}=1.9$. **m** Simulated microscope images of 5CB on periodic annular posts as a function of saddle-splay constant ${K}_{24}/{K}_{22}$ and surface anchoring strength. The parameter pairing that best agrees with experiment is highlighted with a red box

To achieve large-scale symmetry breaking, we consider a system with richer patterning topology: a square array of tangentially touching annuli (Fig. 5a). This was fabricated using the same materials, SU8 and DMOAP, as before. The annuli have inner and outer diameters of 10 and 20 μm, respectively. For annuli height of 0.2 μm, +1 defects are no longer apparent. Instead, POM imaging reveals an intriguing two-brush texture that breaks the polar symmetry of the pattern, suggestive of half-charge defects on the interior of each annulus and on the edge of each four-star shaped gap between adjoining annuli (Fig. 5b). Further, since the annuli are adjoining, this broken symmetry extends across multi-annulus domains.

This highly unusual texture is explained by the simulation results of Fig. 5, which show how, for this feature height, saddle-splay-stabilized −1/2 defects spontaneously break the fourfold symmetry of the pattern design. The calculated microscope image, Fig. 5c, corresponding to structures with *W* = 8 μJ/m^2^ and ${K}_{24}/{K}_{22}=1$, agrees well with experiment. Figs. 5d–l show calculated structures with constant *W* = 8 μJ/m^2^, but varying ${K}_{24}/{K}_{22}$, confirming that the structure adopted strongly depends on the latter. As this ratio is varied, the system adopts an intermediate state 5d-f if ${K}_{24}$ is low, and something analogous to the U state shown in Fig. 5j–l for large ${K}_{24}$.

For the structure whose simulated POM most closely resembles experiment, i.e., Fig. 5g–i, elastic distortion of the LC director field is minimal and the variation required to accommodate the curved boundary conditions accumulates near the defect area. This modest elastic distortion is, though, achieved at the cost of a break in the overall symmetry of the domain—neighboring defects are localized at the same cardinal point as one another. In a further experiment, this same symmetry breaking is also obtained using an array of square annuli of appropriate height (Supplementary Fig. 6)—again, all of the −1/2 defects are located at the same corner of their respective square motif.

### Measurement of ${K}_{24}$

Careful comparison between experimental and simulated textures enables us to resolve compatible values of $W$ and ${K}_{24}$ as before. In Fig. 5m, the simulated POM images are displayed as a function of $W$ and ${K}_{24}$, showing that the experimental texture is only seen for a limited range of parameters. Moreover, we can combine these results with those from circular posts with 5CB as described in Methods to obtain a more robust estimation since each geometry exhibits a very different sensitivity to the material parameters (compare Fig. 3f and Fig. 5m). The annular structure is, as remarked above, sensitive to the ratio of ${K}_{24}/{K}_{22}$. Further, a very weak anchoring scenario is excluded by the circular post structure, because here the director would escape vertically to give an indistinct texture. In Fig. 5m, therefore, we highlight the parameter region that shows greatest consistency with experiment for annuli. Combining the two estimates (Supplementary Fig. 7) yields values of $W=8\pm 3\ \upmu {{\mathrm{J/m}}}^{2}$ and ${K}_{24}/{K}_{22}=1\pm 0.3$. For 5CB, this range of values is consistent with literature, but represents a significant narrowing of the bounds^18,20^.

### Multistable device

**Fig. 6:**
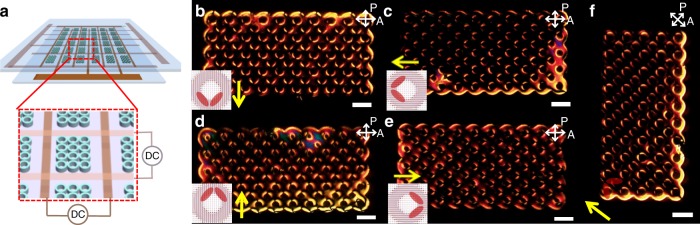
Demonstration of a multistable device. **a** Device schematic showing electrodes and region of annuli. **b**–**e** Set of stable, cardinal states that may be switched into by the application of a small (0.5 V/$\upmu$m) transverse, directional electric field as indicated by each yellow arrow. Each of these states remains stable upon removal of the field and any starting state can be used as a precursor to any chosen new state. **f** By careful tuning of the applied field, the system can also access the four ordinal states, in which the defects reside along system diagonals. All scale bars are $20\ \upmu {\mathrm{m}}$. Insets show the simulation configuration of Fig. 3e, with the distorted regions highlighted

Finally, we leverage this newfound ability to use saddle-splay effects to program symmetry breaking over large domains by creating a multistable, low-voltage, switchable LC device from arrays of annuli (Fig. 6 and Supplementary Fig. 8). To achieve this, we construct a cell with patterned Cu electrodes on each substrate to apply in-plane electric fields (Fig. 6a). Annuli of height 0.2 μm ($5\times 10$ annuli in each array) are patterned on the bottom substrate with the same surface treatment as in Fig. 5. The initial state of such cells is set by the symmetry of the patterned surface, the defects always picking out one of the cardinal compass points, e.g., north-to-south shown in Fig. 6b. Switching between these is then readily achieved by an in-plane electric field (Fig. 6b–e). Response times are measured as described in Methods, and found to be 250 ms (rising) and 400 ms (falling) for 180° switching, and 240 ms (rising) and 410 ms (falling) for 90° switching (Supplementary Fig. 9). Videos of switching between states are included as supplementary materials (see Supplementary Movies 1 and 2). All switching permutations prove straightforward, whether they require rotation of the in-plane director by $18{0}^{\circ }$ (e.g., from Fig. 6b–d) or $9{0}^{\circ }$ (e.g., from Fig. 6b, c). Furthermore, and very unusually for an LC system, the sense of the applied field is significant—it couples to the pattern-scale polarity of the surface state, even though the underlying LC is intrinsically apolar.

Despite being non-optimized, the threshold switching electric field of ${\sim} 0.5\ {\mathrm{V}}/\upmu{\mathrm{m}}$, for all rotation types, is lower than those generally required to achieve LC switching, particularly in multistable systems. This is, presumably, due to the azimuthal degeneracy of the LCs on the isotropic annuli, energy barriers between the states corresponding to the relocation of the topological defects around each annulus. In other multistable LC technologies, switching requires defects to be either created or annihilated, thereby incurring a high energetic cost^11,35^. In contrast, here the $-1/2$ defects are present in all states, stabilizing the domain-wide symmetry against thermal fluctuations and in-plane flow and, so, delivering multi-stability^37^. Indeed, with careful tuning of the applied electric field, the local symmetry breaking can go beyond the four native stable states (the cardinal points), to additionally include further stable states along the four diagonals (the ordinal points) of the arrays of annuli (Fig. 6f). We also note that since the defect structure persists over a range ${K}_{24}$ and $W$ as shown above, there remains room to tune surface properties. Clearly, the scope for further engineering of saddle-splay-stabilized devices goes well beyond the basic demonstrator considered here.

## Discussion

Our results show that saddle-splay effects, routinely suppressed in LC systems, can be realized using substrates that combine topographical and chemical patterning. Indeed, we have demonstrated that such behaviors are actually rather general, being accessible to standard LC materials and across a range of pattern geometries. That said, it is necessary to achieve fine control of pattern height to fully explore the relevant regime. Further, additional physical effects such as order parameter gradients and flexoelectricity may be important for other geometries and surface treatments, and the dynamics of switching behavior between the states observed here remain to be fully understood.

Saddle-splay effects in confined LCs manifest as modifications to the surface-region director field configurations and defects. These include spontaneous emergence of locally chiral and polar arrangements, which offer a route to experimental measurement of the (historically challenging) elastic constant ${K}_{24}$. For patterns such as touching annuli, however, an even more exciting behavior is seen: the emergent symmetry breaking is no longer localized, but extends across domains. Thus, the intrinsically apolar LC adopts surface director field patterns with regular polar defects which couple to directional DC fields. Indeed, when harnessed in this way, they provide the basis for a novel class of octo-stable optical device with low switching fields.

Having demonstrated the novel capabilities opened up by consideration of saddle-splay effects in a basic sandwich-geometry cell, it is exciting to consider the implications these have for other applications. Such ${K}_{24}$ coupling could open up vistas of behavior in LCs with nanoparticle or colloidal inclusions, LCs confined in bicontinuous 3D periodic structures, or LCs containing complex defect arrays. These include routes to controlling complex self aggregation, novel structured dielectric media, and low-energy data storage functionality.

## Methods

### Materials

N,N-dimethyl-n-octadecyl-3-amino-propyltrimethoxysilyl chloride (DMOAP), gamma-butyrolactone and propylene glycol monomethyl ether acetate (PGMEA) were purchased from Sigma-Aldrich and used as received. 4-cyano-4′-pentylbiphenyl (5CB) and 4-cyano-4′-octylbiphenyl (8CB) were purchased from Kingston Chemicals Limited. Negative-tone photoresist, SU8-2, was purchased from MicroChem Corporation, and positive-tone photoresist, S1813, was purchased from Dow Chemical Company.

### Continuum theory simulations

The computational domain is periodic in the $x$ and $y$ directions with side $\Lambda =20\ \upmu{\mathrm{m}}$ and $d=25\ \upmu{\mathrm{m}}$ tall; the post height is $20\ \upmu{\mathrm{m}}$. The free energy to be minimized consists of bulk elastic ${f}_{el}=\frac{1}{2}\left[{K}_{1}{\left(\nabla \cdot {\bf{n}}\right)}^{2}+{K}_{2}{\left({\bf{n}}\cdot \nabla \times {\bf{n}}\right)}^{2}+{K}_{3}{\left|n\times \nabla \times n\right|}^{2}\right]$, surface-like saddle-splay elasticity $-\frac{1}{2}{K}_{24}\left[{\bf{n}}\times \nabla \times {\bf{n}}+{\bf{n}}\nabla \cdot {\bf{n}}\right]\cdot \hat{{\bf{s}}}$ and anchoring contributions $\pm W{\left(n\cdot {{\bf{n}}}_{e}\right)}^{2}/2$ where ${\bf{n}}$ is the cartesian director ${\bf{n}}=({n}_{x},{n}_{y},{n}_{z})$ and $\hat{{\bf{s}}}$ is the outward surface normal. ${K}_{1}$, ${K}_{2}$, and ${K}_{3}$ correspond to splay, twist, and bend deformations, respectively, and ${K}_{24}$ is the saddle-splay constant. $W$ is the anchoring coefficient. The choice of sign in the anchoring term gives either alignment with an easy axis ${{\bf{n}}}_{e}$ if negative or planar degenerate alignment if ${{\bf{n}}}_{e}$ is chosen to be $\hat{{\bf{s}}}$ and if positive. This energy is nondimensionalised and discretized using finite differences onto a $31\times 31\times 30$ grid and a nonuniform grid spacing is used in the $z$ direction, concentrating additional mesh points around the post; to ensure stability on the nonuniform grid first order finite differences are used. The length constraint ${\bf{n}}\cdot {\bf{n}}=1$ is imposed locally by Lagrange multipliers. Grid points inside the posts are omitted and boundary conditions are imposed as follows: on the top substrate, vertical alignment is imposed; on the bottom surface, vertical boundary conditions are imposed outside the posts, and planar degenerate conditions are imposed on the side and top of the posts.

Parameters used are as follows: in the absence of an experimental measurement, the vertical DMOAP anchoring coefficient was chosen by setting the dimensionless parameter $\Gamma =W\lambda /{K}_{1}=1$ yielding $W=3.15\times 1{0}^{-4}\ {\mathrm{J}}{{\mathrm{m}}}^{-2}$, which corresponds to strong anchoring; this is the same order of magnitude to the measurement of $W=1\times 1{0}^{-4}\ {\mathrm{J}}{{\mathrm{m}}}^{-2}$ for 8OCB in DMOAP^38^. In addition, calculations were repeated for values of ${W}_{{\mathrm{DMOAP}}}$ in the range $1-10\times 1{0}^{-4}\ {\mathrm{J}}{{\mathrm{m}}}^{-2}$ and found not to perturb the calculated structures, and hence the measured value of ${K}_{24}$ significantly. For $SU8$ anchoring coefficients on the interval $W=[3,32]\ \upmu{\mathrm{J}}/{{\mathrm{m}}}^{2}$ are used, spanning a similar range to experimental measurements of 5CB alignment on other surfaces that promote weak zenithal anchoring such as weakly rubbed nylon^39,40^, photoalignment layerss^39^, rubbed polyimide^41^, and silicon oxide^42^ (see Supplementary Table 1). We also note that no evidence of elastic instabilities that might occur for sufficiently weak anchoring were observed. Experimentally measured liquid crystal elastic constants are used based on room temperature^36^: for 5CB, ${K}_{1}=6.3\ {\mathrm{pN}}$, ${K}_{2}=4.3\ {\mathrm{pN}}$, ${K}_{3}=9.6\ {\mathrm{pN}}$ are used; for 8CB ${K}_{1}=7.1\ {\mathrm{pN}}$, ${K}_{2}=3.3\ {\mathrm{pN}}$, ${K}_{3}=10.7\ {\mathrm{pN}}$. For both materials, ${K}_{24}$ is varied over the entire interval allowed by the Ericksen inequalities.

The energy is minimized by a gradient descent scheme with line searches and adaptive step size and the process repeated until the energy converges to the relative change per iteration is <$1{0}^{-6}$. Sample configurations, broken down by splay, twist and bend energy are displayed in Supplementary Figs. 2 and 3.

### Preparation of surface topographical patterns

Surface topographical patterns were fabricated from photoresist SU8 using conventional photolithography (Supplementary Fig. 1a). Glass substrates were pre-cleaned by rinsing with acetone three times, followed by drying with an air gun. For $0.2\ \upmu$m patterns, commercial photoresist SU8-2 was diluted four times with gamma-butyrolactone, followed by spin-coating (4000 rpm for 40 s) on a clean glass substrate. The sample was then prebaked at 95 Â°C for 1 min, and exposed to 365 nm UV light (Newport model 97436-1000-1, Hg source) through a photomask with a dosage of 200 mJ/cm^2^. After post-baking at 95 °C for 1 min, the sample was developed in PGMEA to obtain the final SU8 pattern. The $2\ \upmu$m patterns were prepared through a similar procedure with undiluted SU8-2 and a slightly lower spin-coating velocity at 2000 rpm for 40 s.

### Surface chemistry modification

In all, 3 vol% DMOAP solution was prepared in a mixture of water/ethanol (1:9 v/v). For non-patterned glass substrates, they were immersed in DMOAP solution for 30 min. For SU8 patterned glasses, DMOAP treatment was varied to find the optimal condition to generate homeotropic anchoring in non-patterned region while maintaining SU-8 patterned region planar. The DMOAP treated substrates were washed with deionized (DI) water three times and baking at 110 Â°C in a convection oven for 1 h.

### Water contact angle measurement

To monitor the DMOAP coating kinetics, flat SU-8 film was prepared without surface pattern as a comparison to glass substrate in the reaction with DMOAP (Supplementary Fig. 1c). Both the SU-8 film and glass substrate were treated by DMOAP solution, and water contact angle was measured as a function of DMOAP treatment time. For water contact angle measurement, a 5 μL water droplet was placed on the sample surface from Ramé-Hart standard automated goniometer (model 200) using the sessile drop method. For each water contact angle reported, it was averaged over three measurements at different locations of the sample.

### Preparation of liquid crystal cells

Liquid crystal cells were constructed from one glass slide with chemical patterns and the other one as DMOAP treated non-patterned substrate. The thickness of the LC cells was controlled using a Mylar spacer (~5-μm thick). LCs were then infiltrated into the cells through capillary filling. To remove the mechanical and thermal history of the LC, samples were heated on a hot stage to the isotropic phase (35 Â°C for 5CB and 42 Â°C for 8CB) and maintained for 1 min, followed by slowly cooling down (1 Â°C/min) to the desired temperature for the anchoring study with optical microscopy (Olympus BX61).

### Chirality calculations

For a given image the circular region of interest (ROI) on top of the post is identified and denoted $I$ (see Supplementary Fig. 4). The ROI is reflected about an arbitrary axis passing through the center of the post to produce a new image $I^{\prime}$ and the chirality about this axis is calculated $\chi ={\chi }_{0}{\sum }_{ij}(I-I^{\prime} )/{\sum }_{ij}$ where the sums are over pixels in the region of interest. The axis which produces the smallest value of $\chi$ is then chosen from 100 orientationally equally spaced axes (see Supplementary Fig. 4b, c). The normalization constant ${\chi }_{0}$ is then chosen to map observed values onto the range $[0,1]$.

### Parameter estimation

The saddle-splay constant and SU8 anchoring strength are estimated by comparing simulated POM images across a range of parameters to experimentally observed structures, yielding feasible regions highlighted with blue dash lines in Fig. 3e–g and Supplementary Fig. 7. For pinwheel structures on circular posts, the chirality $\chi$ of both experimental and simulated structures is calculated as described above. Experimental images give distributions as shown in Fig. 3d from which the mean experimental chirality is calculated as $\bar{\chi }$ for a given post height. We then select the region of parameter space where the simulated $\chi$ is within 1 standard deviation of $\bar{\chi }$; these regions are highlighted in Fig. 3f, g and Supplementary Fig. 7a. For boojum and uniform structures on circular posts, the region where the experimentally observed boojum state is observed is highlighted in Fig. 3e. For the annular posts, the region of parameter space where the simulated structures reproduce the two-brush texture observed experimentally. This region is highlighted in Supplementary Fig. 7b. The intersection of the three feasible regions of parameter space for 8CB on circular posts is then taken and outlined with red solid lines on Fig. 3e–g; the corresponding region for 5CB is shown in Supplementary Fig. 7 using both circular and annular posts. Our final quoted values of ${K}_{24}$ for 5CB represent the extent of the feasible region in Supplementary Fig. 7.

### Preparation of multistable device

To study multi-stability of our annuli device, a LC cell was constructed by one surface with chemical patterns, and the other surface with a patterned Cu electrode that applied the demanding in-plane electric field for LC director switching (see schematic in Fig. 6 and Supplementary Fig. 8).

To prepare patterned Cu electrodes, pre-cleaned microscope glass slides were sputtered with Cu for 1 min to obtain a thickness ${\sim}80$ nm. Positive-tone photoresist S1813 was then spin-coated (2000 rpm for 40 s) on the Cu-sputtered glass, followed by pre-baking on a hot plate at 110 Â°C for 1 min. Next, the sample was exposed to 365 nm UV light at the Newport Hg light source though a photomask at a dosage of 200 mJ/cm^2^, followed by developing in MF-319 solution to obtain the electrode pattern. The sample was then etched in 0.3% Cu etchant and rinse with DI water. For a better quality of DMOAP coating, the sample was also coated with a thin layer of SiO${}_{2}$ though a chemical vapor deposition (CVD) process: the patterned Cu substrate was kept in a desiccator under vacuum in the presence of silicon tetrachloride (SiCl${}_{4}$, 0.2 mL) for 10 min. Then the sample was treated with water vapor in a humidity chamber (humidity ${\sim}90$%) for 10 min. After washing with ethanol and DI water, respectively, for three times, the patterned Cu electrode was coated with DMOAP, followed by drying by an air gun and baking at 110 Â°C in a convection oven for 15 min.

To construct a multistable LC cell, the chemical pattern and the Cu electrode were aligned under an ABM mask aligner, with the thickness of the LC cell controlled by a Mylar spacer (~25-μm thick). 5CB was then infiltrated into the LC cell through capillary filling. To remove the mechanical and thermal history of 5CB, samples were heated on a hot stage to 35 Â°C to the isotropic phase and maintained for 1 min, followed by slowly cooling down (1 Â°C/min) to room temperature for the anchoring study with optical microscopy.

### Multistable switching

To study multi-stability, an in-plane DC electric field was applied through the Cu electrode and maintained for ${\sim}1$ s, following by characterization of LC texture under a POM. The voltage of the applied electric field was increased from 10 to 110 V at an interval of 10 V. A voltage of 100 V (electric field strength = 0.5 V/μm) was identified as the minimal requirement for switching the multistable device.

### Response time measurement

Dynamic responses of the device are measured using a white light emitting diode (LED) light and a photoresistor (purchased from Elegoo Inc.) in conjunction with the driving circuits (parts also purchased from Elegoo Inc.). The device is placed between two crossed polarizers and transmittance curves were obtained under the applied voltage with the amplitude of 100 V using a DC power supply (HP 6515A). The rising and falling times were estimated from the normalized transmittance curves (from 10 to 90% and vice versa) and they were measured as 250 and 400 ms for 180° switching and 240 and 410 ms for 90° switching, respectively.

### Characterization

Liquid crystal alignment was characterized by an Olympus BX61 motorized optical microscope with crossed polarizers using CellSens software. Sample annealing was carried out on a Mettler FP82 and FP90 thermo-system hot stage under ambient condition. SEM imaging was performed on a dual beam FEI Strata DB 235 Focused Ion Beam (FIB)/SEM instrument with 5 KV electron-beam. AFM imaging was performed on a Bruker Icon AFM.

## Supplementary information

Supplementary Information

Description of Additional Supplementary Files

Supplementary Movie 1

Supplementary Movie 2

## Data Availability

The data that support the findings of this study, as well as the models used in our calculations, are available from the corresponding author upon reasonable request.

## Supplementary information

**Supplementary information** available for this paper at 10.1038/s41467-019-13012-9.

## Code availability

Codes used to perform the modeling are available from the corresponding authors upon reasonable request.
