# Supplementary material for: Programming emergent symmetries with saddle-splay elasticity
Source: Nat Commun. 2019 Nov 8;10:5104. doi: 10.1038/s41467-019-13012-9 (PMC6841980; doi:10.1038/s41467-019-13012-9)
Supplement: Supplementary file 1 — Supplementary Information [file 41467_2019_13012_MOESM1_ESM.pdf]

Programming Emergent Symmetries with Saddle-Splay Elasticity

Yu Xia *et al.*

SUPPLEMENTARY FIGURES

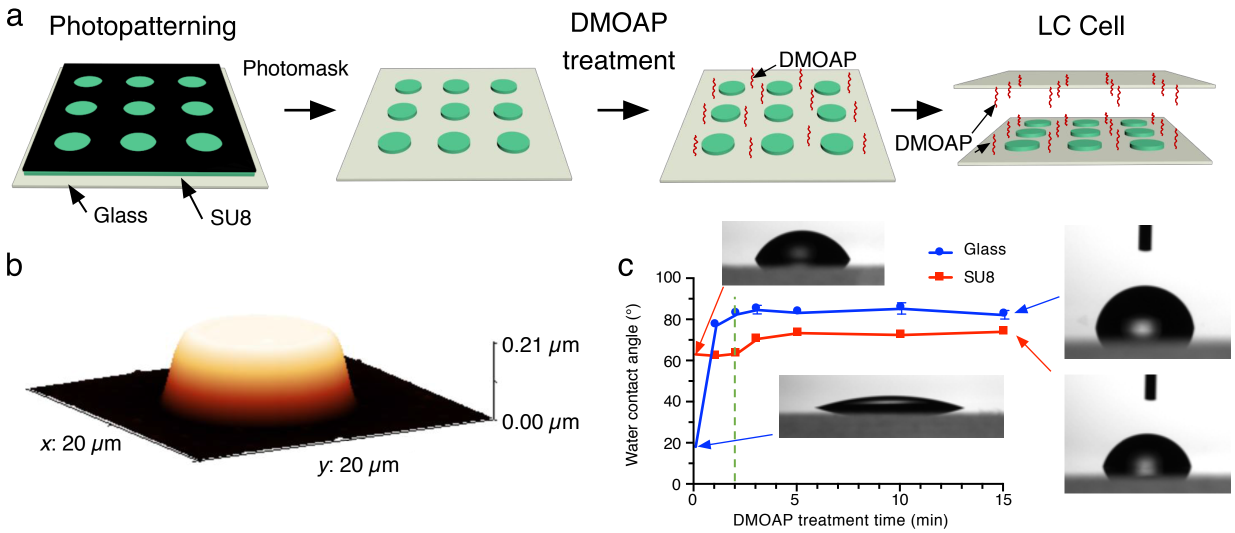

Supplementary Figure 1: Photopatterning and surface treatment. **a** Photopatterning and surface treatment. a Schematic illustration of the sample preparation process. Different color indicates different material: grey – photomask, green – SU8, red – DMAOP. Mono-layer of DMOAP is coated on glass through surface treatment in solution. The final LC cell is constructed with one chemically-patterned glass (bottom) and the other DMOAP-coated nonpatterned glass (top). **b** AFM measurement of a single SU8 circle on glass. **c** Water contact angle measurement of glass and SU8 with different DMOAP treatment time. Insets are optical microscopy (OM) images of water drops on the sample surface. Green dotted line indicates the optimized DMOAP treatment time (2min). Right panels show OM images of water drops on SU8 (top panel) and glass (bottom panel) after 2min DMOAP treatment.

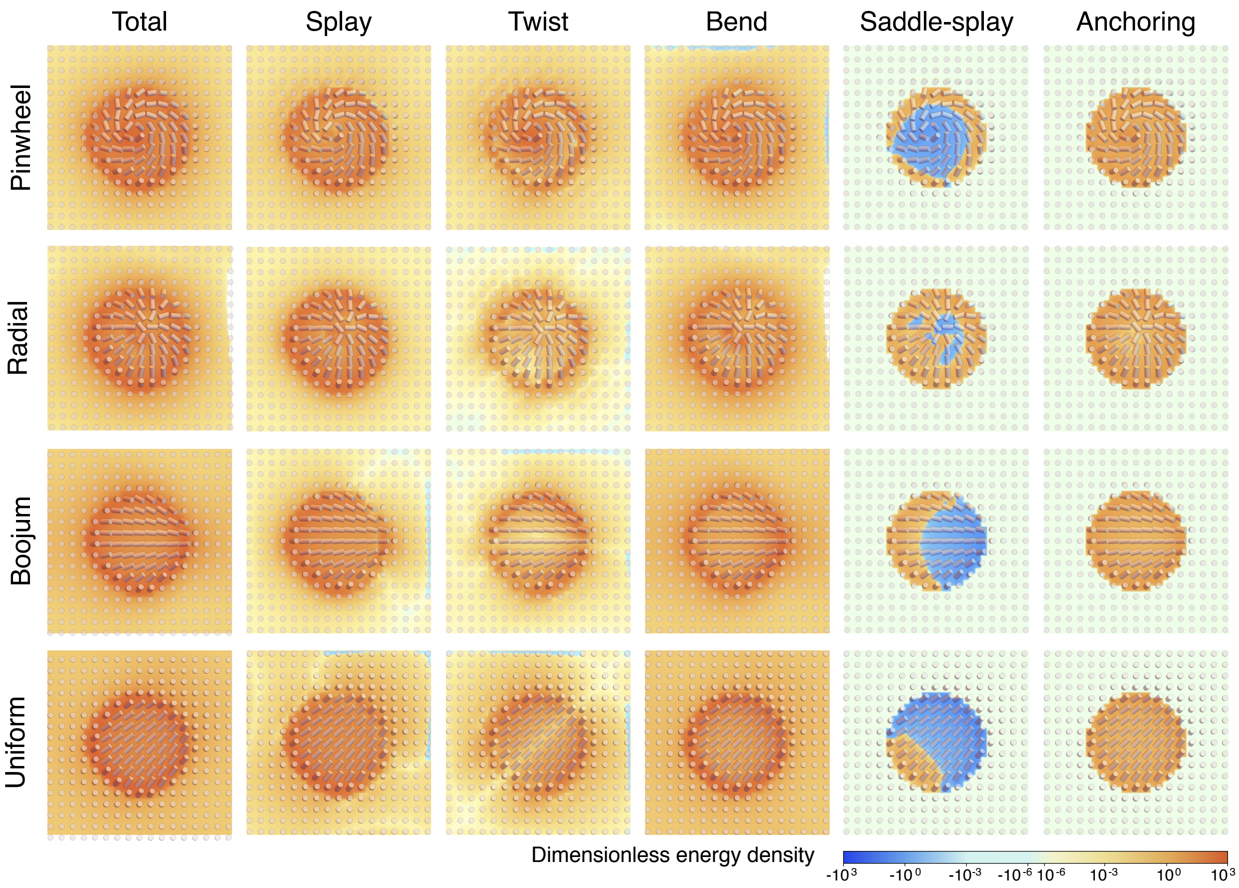

Supplementary Figure 2: Simulated contributions to the energy of 5CB on top of circular posts. All modes of elastic deformation and surface anchoring in four types of possible configurations are plotted in colored energy density maps.

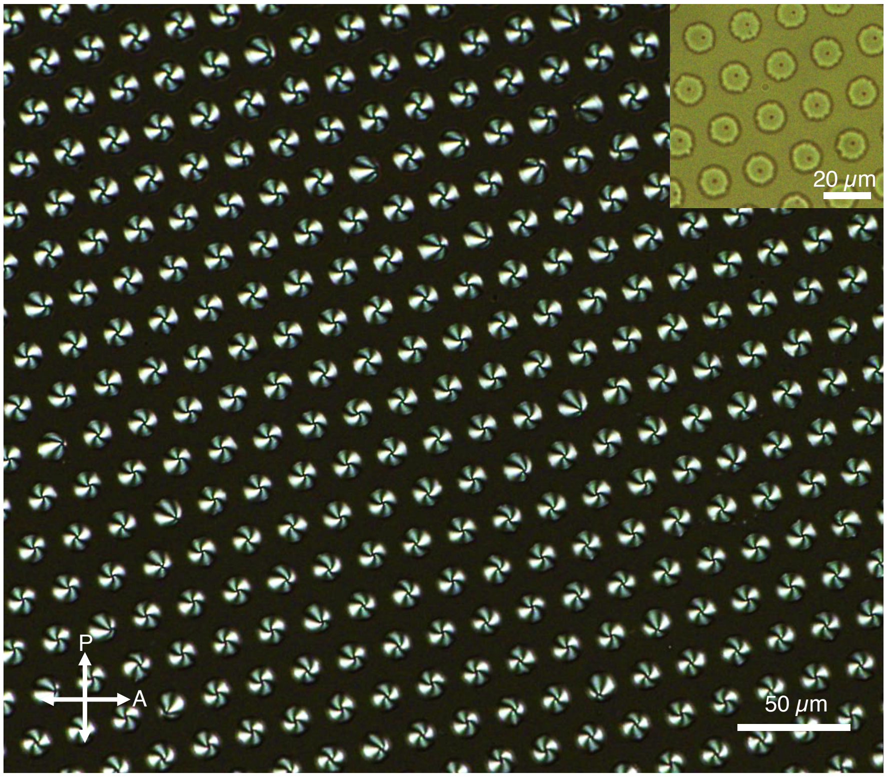

Supplementary Figure 3: Polarized optical microscopy (POM) images of 5CB on circular posts with height of 0.2 µm. Chiral symmetry breaking with +1 defects are seen on top of the posts over a large area. Inset: Bright field (BF) image showing the topological defects of 5CB (black dots).

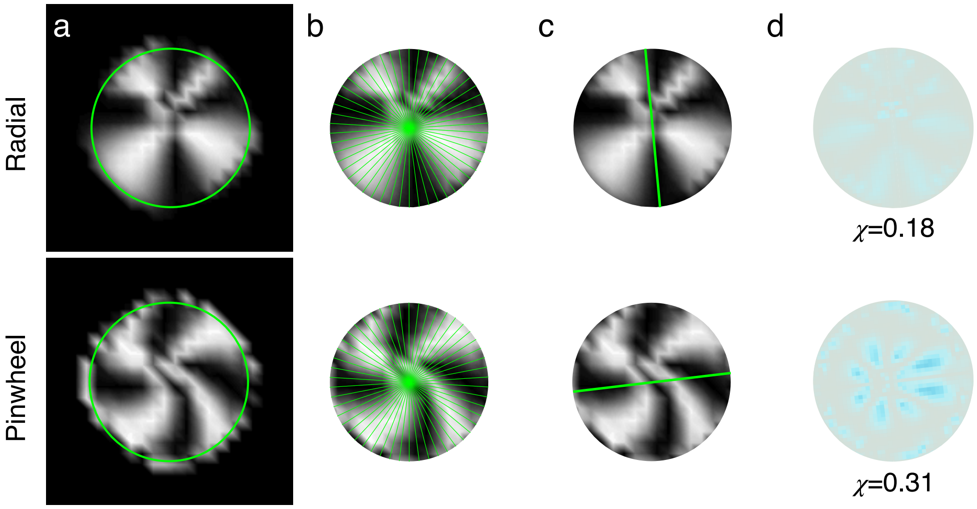

Supplementary Figure 4: Chirality measurement χ from simulation. a Simulated POM images of the radial (top) and pinwheel (bottom) configurations overlaid with a circle showing the region used to determine the image chirality. **b** The region of interest is isolated and the reflection symmetry is calculated about 100 bisecting lines (only 25 lines shown). **c** The most favorable symmetry axis is chosen from all the trials. **d** Heat map of the pixel intensity difference between the original and reflected images; cyan regions indicate a norm close to unity. The integral of this difference, normalized by area, gives us χ.

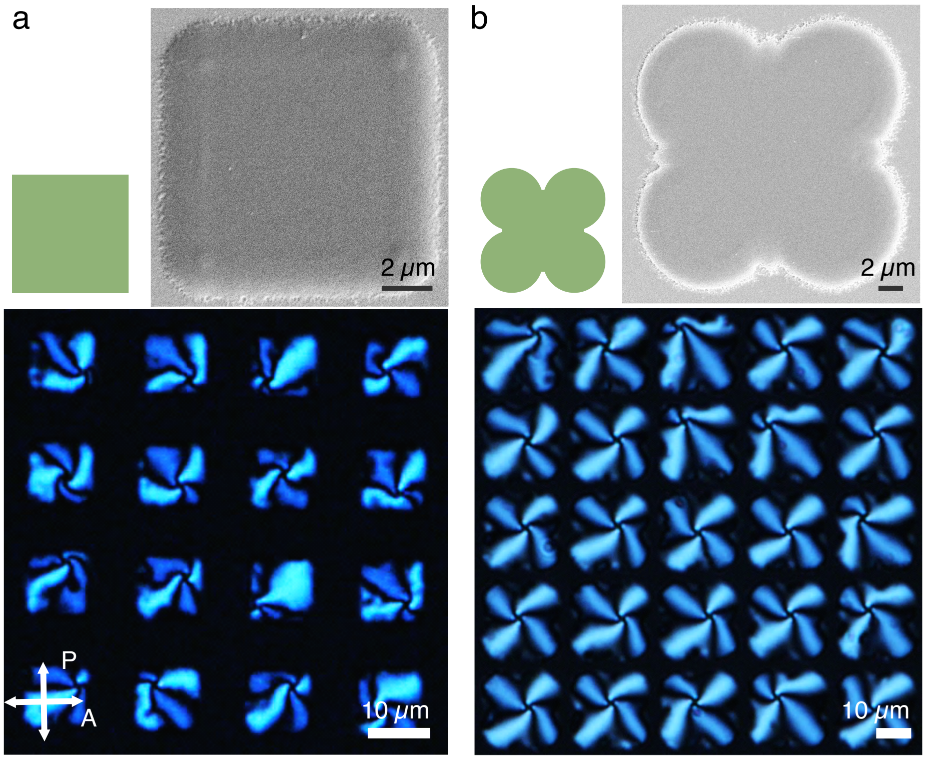

Supplementary Figure 5: 5CB on posts with different shape. Top panels: designs and SEM images of the patterns, and bottom panels: POM images of 5CB on posts with shape of **a** square, **b** four-panel clover.

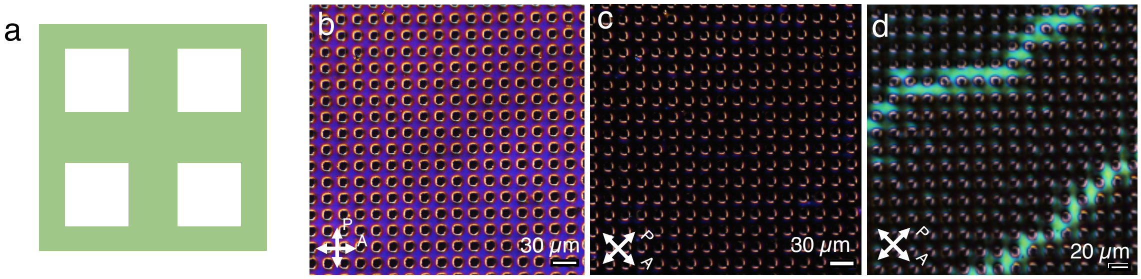

Supplementary Figure 6: 5CB on array of square washers. **a** Schematic illustration of the square washers. **b-c** POM images of 5CB on the array of square washers at 0° (b) and 45° (c) polarizer angle. **d** POM image shows three meta-stable LC alignment. The blue regions demonstrate the grain boundaries between different alignments.

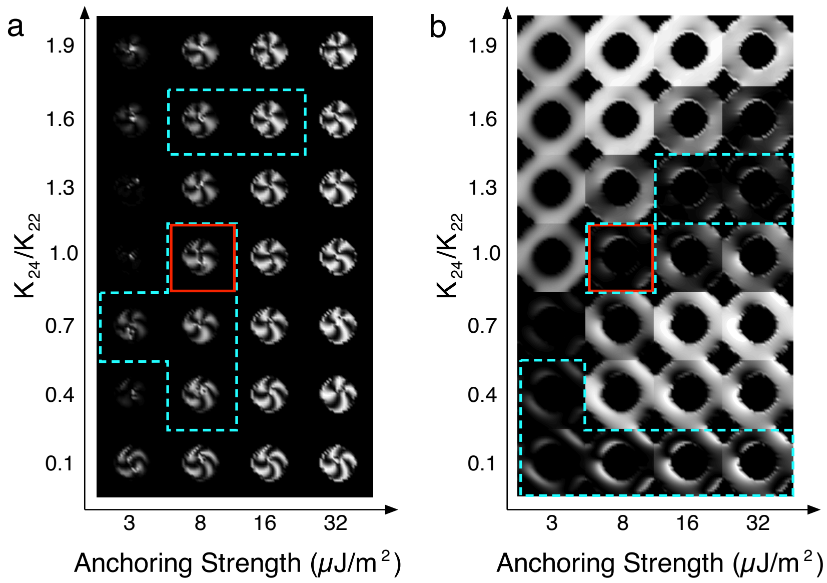

Supplementary Figure 7: Simulated POM images of 5CB configuration as a function of saddle-splay constant (K_{24}/K_{22}) and SU8 surface anchoring strength. **a** Structures for circular posts: Blue dash region indicates structures with chirality χ within 1 standard deviation of the experimentally observed average $\bar{\chi}$ are highlighted. **b** Structures for annular posts where blue dash region indicates the region of parameter space where the experimentally observed two-brush texture is the ground state. The intersection of both both circular and annular feasible feasible regions is highlighted with a red box.

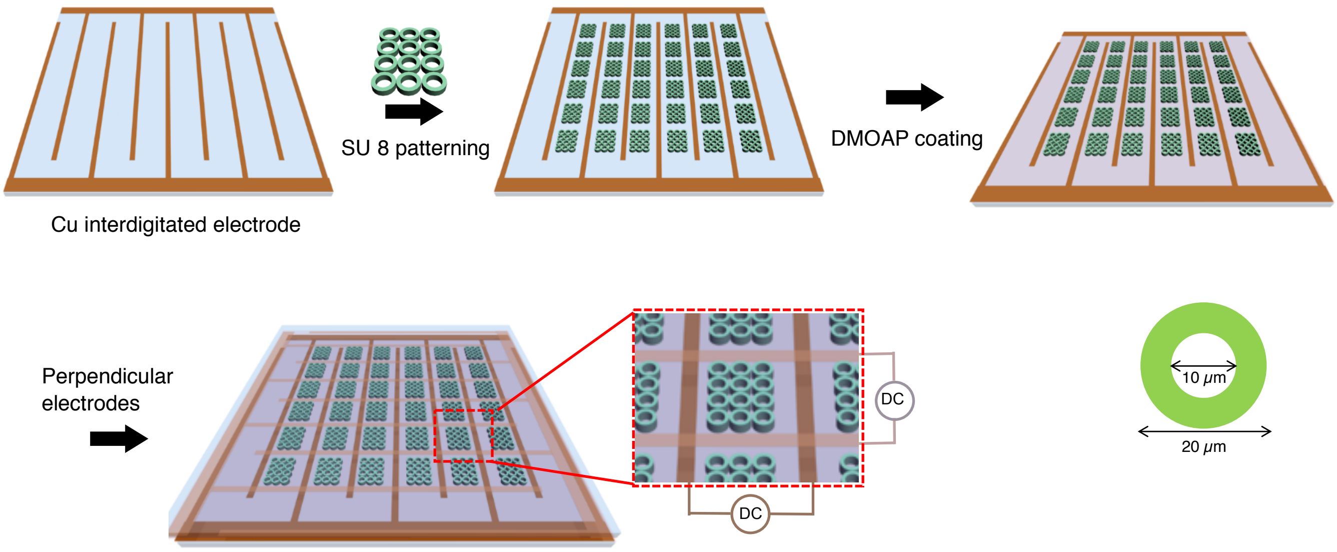

Supplementary Figure 8: Fabrication of multistable device. Periodic annular posts were photo-patterned between the interdigital Cu electrodes. Then, the substrate was chemically treated by DMOAP. The DMOAP treated patterned electrodes were orthogonally sandwiched with ~25μm gap, in which infiltrated LC can be switched in four ways by the application of a transverse DC field.

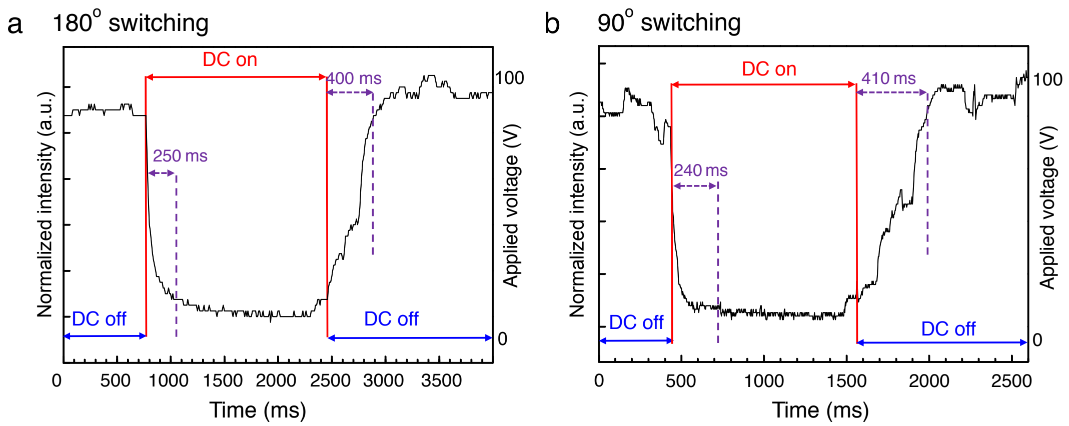

Supplementary Figure 9: Response times of the multistable device. a For 180° switching, the measured rising and falling times are 250 and 400 ms, respectively. **b** For 90° switching, the measured rising and falling times are 240 and 410 ms, respectively.

SUPPLEMENTARY TABLES

LC	Alignment Film	W_θ	Technique	Ref.
MBBA	Surfactant	$1.17 \times 10^{-5} Jm^{-2}$	Magnetic switching	Appl. Phys. Lett. **43**, 62 (1983)
5CB	SiO	$4 \times 10^{-5} Jm^{-2}$	Electric switching	J. Appl. Phys. **57**, 4520 (1985)
5CB	Rubbed Polyimide	4-20 $\times 10^{-4} Jm^{-2}$	Electric switching	J. Appl. Phys. **86**, 4199 (1999)
5CB	Rubbed Polyimide (NASA LARC CP1)	$5 \times 10^{-5} Jm^{-2}$		
E7	Rubbed Polyimide	$1.2 \times 10^{-4} Jm^{-2}$	Electric switching FLGM	J. Appl. Phys. **87**, 2726 (2000)
E7	Lecithin	$4.6 \times 10^{-4} Jm^{-2}$	Electric switching FLGM	J. Appl. Phys. **88**, 6175 (2000)
5CB	Moderately rubbed Nylon	$2.9 \times 10^{-5} Jm^{-2}$	Dynamic Light Scattering	Phys. Rev E **65**, 041712 (2002)
5CB	Strongly Rubbed Nylon	$1 \times 10^{-4} Jm^{-2}$		
5CB	Photoalignment layer	$1.2 \times 10^{-6} Jm^{-2}$		
5CB	Rubbed Nylon	5-15 $\times 10^{-6} Jm^{-2}$	Dynamic Light Scattering	Phys. Rev E **68** 031704 (2003)
8OCB	DMOAP	$1 \times 10^{-4} Jm^{-2}$	Dynamic Light Scattering	Liq. Cryst. **33** (5) 581 (2006)

Supplementary Table I: Selected literature measurements of polar anchoring energy W_θ.
