# Supplementary material for: Programming emergent symmetries with saddle-splay elasticity
Source: Nat Commun. 2019 Nov 8;10:5104. doi: 10.1038/s41467-019-13012-9 (PMC6841980; doi:10.1038/s41467-019-13012-9)
Supplement: Supplementary file 2 — Description of Additional Supplementary Files [file 41467_2019_13012_MOESM2_ESM.pdf]

Description of Additional Supplementary Files

Supplementary Movie 1: 90° switching. -1/2 defects array in annuli pattern, which is switched into by the application of a small (0.5 V/mm) transverse, 90°-directional electric field as indicated by each yellow arrow in the movie.

Supplementary Movie 2: 180° switching. -1/2 defects array in annuli pattern, which is switched into by the application of a small (0.5 V/mm) transverse, 180°-directional electric field as indicated by each yellow arrow in the movie.
